# Mutations of *SETBP1* and *JAK3* in juvenile myelomonocytic leukemia: a report from the Italian AIEOP study group

**DOI:** 10.18632/oncotarget.8016

**Published:** 2016-03-09

**Authors:** Silvia Bresolin, Paola De Filippi, Francesca Vendemini, Mirko D'Alia, Marco Zecca, Lueder H. Meyer, Cesare Danesino, Franco Locatelli, Riccardo Masetti, Giuseppe Basso, Geertruy te Kronnie

**Affiliations:** ^1^ Department of Women's and Children's Health, Laboratory of Oncohematology, University of Padova, Padova, Italy; ^2^ Department of Molecular Medicine, University of Pavia, Pavia, Italy; ^3^ Oncologia ed Ematologia Pediatrica “Lalla Seràgnoli”, University of Bologna, Ospedale S. Orsola Malpinghi, Bologna, Italy; ^4^ Oncoematologia Pediatrica, Istituto di Ricovero e Cura a Carattere Scientifico (IRCCS), Fondazione Policlinico San Matteo, Pavia, Italy; ^5^ Department of Pediatrics and Adolescent Medicine, Ulm University Medical Center, Ulm, Germany; ^6^ Department of Pediatric Onco-Hematology, IRCCS Ospedale Pediatrico Bambino Gesù, Roma, University of Pavia, Pavia, Italy

**Keywords:** JMML, SETBP1, JAK3, murine model

## Abstract

Juvenile myelomonocytic leukemia (JMML) is a rare aggressive disease of early childhood. Driver mutations in the Ras signaling pathways are a key feature of JMML patients. Mutations in *SETBP1* and *JAK3* were recently identified in a subset of JMML patients characterized by poor prognosis and progression of disease. In this study, we report the results of a screening for mutations in *SETBP1* and *JAK3* of a cohort of seventy Italian patients with JMML, identifying 11.4% of them harboring secondary mutations in these two genes and discovering two new mutations in the SKI domain of *SETBP1*.

JMML xenotransplantation and colony assay provide an initial understanding of the secondary nature of these events occurring in early precursor cells and suggest a different propagating capacity of clones harboring particular mutations.

## INTRODUCTION

Juvenile myelomonocytic leukemia (JMML) is a rare aggressive leukemia of early childhood, characterized by excessive proliferation of monocytic and granulocytic cells, which can infiltrate organs, including spleen, liver, gastrointestinal tract, and lung. [1]. Around 90% of newly diagnosed JMML patients are characterized by driver mutations in Ras signaling pathway genes including *PTPN11*, *KRAS*, *NRAS*, *CBL* and *NF1* [2, 3]. Using whole exome sequencing, secondary mutations in *SETBP1* and *JAK3* were recently found in 17% of children with JMML, conferring poor prognosis to mutated patients [4]. Moreover, for *SETBP1* an increased frequency of mutations was discovered (30.3%), considering also subclonal mutations identified using droplet digital polymerase chain reaction (ddPCR) [5].

*SETBP1* is a proto-oncogene over-expressed in myeloid progenitor cells leading to enhance self-renewal capacity through direct transcriptional activation of *HOXA9* and *HOXA10* [6]. SETBP1 is known to bind the SET nuclear oncoprotein [7] with an inhibitory effect on protein phosphatase type 2a (PP2A) [8].

Although the role of *SETBP1* in leukemogenesis remains elusive, deleterious mutations of *SETBP1* have been reported to occur with high frequency in adults with atypical chronic myeloid leukemia (aCML) [9], chronic myelomonocytic leukemia (CMML) and secondary acute myeloid leukemia (AML) [10–13]; by contrast, mutations have been found only rarely in pediatric myeloid malignancies. *JAK3* encodes for a member of the Janus family of tyrosine kinases essential for signal transduction through the JAK/STAT pathway shown to be crucial for the development of lymphoid cells, in particular mature T and Natural Killer (NK) lymphocytes [14]. Functional *JAK3* mutations in the PTK (Pseudokinase) domains have been identified in acute megakaryoblastic leukemia (AMKL), T-cell prolymphocytic leukemia and NK T-cell lymphoma [15].

Here, we report the incidence of mutation events in the functional domains of *SETBP1* and *JAK3* in a cohort of JMML AIEOP (Italian Pediatric Hematology and Oncology Association) patients, together with data pointing to a propagating capacity in mutated clones.

## RESULTS AND DISCUSSION

**Figure 1:**
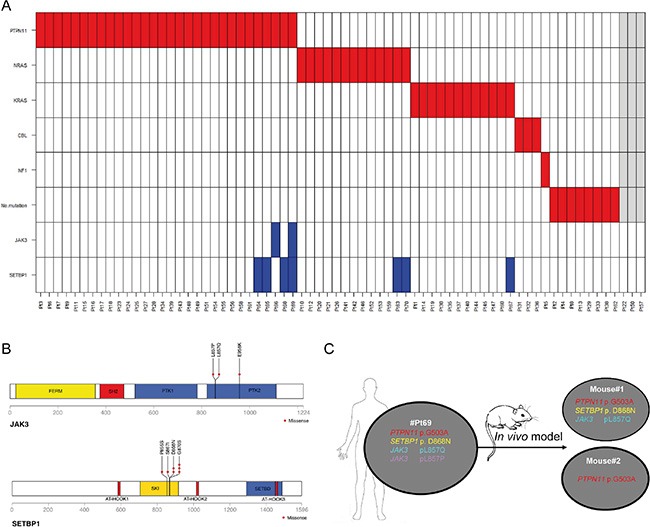
(**A**) Mutation profile of 70 JMML patients. Sixty-seven patients were screened for *PTPN11*, *NRAS*, *KRAS*, *CBL* mutations, clinical signs of NF1 and secondary *SETBP1* and *JAK3* mutations. Asterisk indicates the presence of a heterozygous *CBL* mutation. In grey are 3 patients for which the driver mutation status is not known but were included in the *SETBP1* and *JAK3* mutation screening. (**B**) Distribution of missense alterations at the functional domains of JAK3 and SETBP1 proteins. Altered amino-acids identified in our cohort are highlighted: plus sign in JAK3 p959 amino-acid indicates that the amino-acid was altered only at the genomic level; red arrows point to the novel p.P855S and p.S867I mutations in the SKI domain of SETBP1. (**C**) Scheme of the *in vivo* assay indicating the patient sample xenografted. SH2: Src homology 2 domain; PTK1 and PTK2: pseudo-kinase domain; SKI: v-ski sarcoma viral oncogene homolog domain; SETBD: SET-binding domain.

Known driver mutations in the Ras signaling pathway were found in 84% of 70 patients analyzed in this study; 36% were mutated in *RAS* (*NRAS* or *KRAS*), 43% in *PTPN11*, 4.2% in *CBL* and one patient had a diagnosis of NF1 (Neurofibromatosis syndrome type1), whereas 11% of patients did not harbor any mutations of the aforementioned genes (Figure 1A).

Mutation hotspot regions covering the regions encoding for the SKI homologous domain of SETBP1 and for the PTK domain of JAK3 were included in the mutation analysis (Figure 1B). Seven out of 70 patients (10%) carried *SETBP1* mutations and 2 out of 70 (2.8%) mutations in *JAK3*. One patient harbored a single *JAK3* mutation and the other one carried mutations in both *SETBP1* and *JAK3* (Figure 1A) in two different *JAK3* mutated clones (p.L857Q and p.L857P).

The two *JAK3* mutations, both predicted to be deleterious by SIFT and PolyPhen bioinformatics tools, were found in distinct sub-clones. Among *SETBP1* mutations, two new mutations in the SKI domain (p.P855S, p.S867I), not previously described, were identified. These new mutations cluster in the evolutionary, highly conserved region that bears also the known mutations of *SETBP1* and were predicted to be deleterious and damaging for the protein function by SIFT and PolyPhen-2 (Figure 1B, Supplementary Table 1). The new mutations cluster in the same SKI-homologous domain of SETBP1 protein as previously reported mutations, supposedly altering the protein function by decreasing its stability [9].

All mutations identified here were detected at genomic and transcriptome level, except for the mutation at JAK3 p.E958K. Indeed, #Pt66 showed a genomic mutation and only wild-type expression of *JAK3* at diagnosis (Supplementary Figure S1). Moreover, the BM sample at relapse of this patient did not show any sign of the genomic *JAK3* alteration, indicating lack of clonal propagation of the *JAK3* mutated clone (Supplementary Figure S1). The wild-type *JAK3* expression at diagnosis and the absence of the mutation at relapse suggest that a functional role at the protein level cannot be attributed to this mutation. These observations indicate that, at least in our cohort of patients, the role of mutated *JAK3* in JMML is very limited.

In our cohort of JMML patients, secondary mutations of *SETBP1* and *JAK3* were exclusively found in association with mutated *PTPN11* or *RAS* (*p =* 0.0027 and *p =* 0.002, respectively) (Table 1) and correlated with older age (median 56.8 months vs 23.67 months, *p =* 0.037, Table 1). This observation is in line with a general age-related accumulation of mutations and with the low average number of mutations per sample in JMML patients, compared to those reported in other human cancers, being JMML a disease of young patients [4].

**Table 1:** Clinical and genetic characteristics of the patients included in the study

Characteristic	Total cohort *n* = 70	Secondary mutations		*P*-value
		Yes (*n* = 8)	No (*n* = 62)	
**Gender (M/F)**	46/24	4/4	42/20	NS
**Median age at diagnosis (months)**	23.9 (2–210)	56.8 (5–210)	23.67 (2–158)	0.0374
**Genetic mutation in RAS pathway (67 available)**				
***PTPN11***	30	5 (13%*SETBP1*–3% *JAK3*)	25	0.0027
***NF1***	1	0	1	NS
***NRAS***	13	2 (15% *SETBP1*)	11	0.002*
***KRAS***	12	1 (8% *SETBP1*)	11	NS
***CBL***	3	0	3	NS
**No mutations**	8	0	8	
**Secondary genetic mutation**				
***SETBP1***	7	7	0	
***JAK3***	2	2	0	
**Cytogenetics (53 available)**				
**Normal karyotype**	41	6	35	NS
**Monosomy 7**	10	1	9	NS
**Other abnormalities**	2	0	2	NS
**WBC at diagnosis ×10^9^, median (range)**	32.4 (2.4–226)	44.5 (2.4–71)	27.5 (7.7–226)	NS
**Monocyte at diagnosis ×10^9^, median (range)**	4.4 (1.1–38)	3.8 (2.8–6.4)	4.6 (1.1–38)	NS
**HbF% at diagnosis median (range)**	10 (0.6–80)	8.5 (2.1–32.4)	7.6 (0.6–80)	NS
**PLT at diagnosis × 10^9^, median (range)**	48 (5–192)	65.5 (25–137)	43.3 (5–192)	NS
**HSCT (yes/no)**	62/8	8/0	54/8	-
**Alive/dead**	47/23	3/5	44/18	-

To compare the frequency of mutations with that of other clinical and biological parameters, categorical variables were analyzed using Fisher's exact test and continuous variables with Mann-Whitney *U* test. HbF: hemoglobin F; HSCT hematopoietic stem cells transplantation; PLT platelets; WBC white blood cells.

* *p*-value was calculated considering NRAS and KRAS as a single variable.

White blood count (WBC), monocyte count, platelet count and fetal hemoglobin levels (HbF) did not correlate with the presence of secondary mutations in univariate analysis (Table 1). Survival analysis showed a 10-year OS probability of 0% for patients with secondary mutations and of 60% (SE = 7.5) for patients without mutations in *SETBP1* and *JAK3* (*p =* 0.3) (Supplementary Figure S5).

To evaluate the functional importance of co-occurring mutations in JMML, in particular the *PTPN11* and *SETBP1* mutations, we tested them in a colony assay as previously reported [16]. In this assay, patient cells (*n =* 2) were differentiated *in vitro* into distinct lineages and subsequently tested for the permissiveness of these two mutations. All colonies of immature (LTC-IC) and more mature (BFU-E, CFU-GM and B- and T-lineage) hematopoietic cell types were positive for the presence of both mutations, as revealed by Sanger sequencing except for the colonies of PHA-activated T-cells. Like *PTPN11*, also *SETBP1* mutations are apparently a hit in hematopoietic progenitor cells even if the *SETBP1* mutation is secondary to the *PTPN11* mutation (Supplementary Table 3).

To assess the *in vivo* propagating capacity of cells harboring *JAK3* and *SETBP1* mutations, mononuclear cells isolated from the spleen of #Pt69 harboring PTPN11^p.G503A^, SETBP1^p.D868N^ and JAK3^p.L857Q, p.L857P^ mutations were intravenously transplanted (10^7^ per transplant) in two NSG (NOD SCID gamma) mice (Figure 1C). Nine months after transplantation, mice were sacrificed and both bone marrow (BM) and spleen cells were analyzed by flow-cytometry for huCD45/huCD33 markers (being positive at diagnosis). By this method, engraftment of huCD45/huCD33 cells was observed in the BM of mouse #1, whereas flow-cytometry did not detect huCD45/huCD33 cells neither in the spleen nor in BM of mouse #2 (Supplementary Figure S2). Ultra-deep 454 sequencing analysis (Roche Applied Science, Penzberg, Germany), using human-*PTPN11* specific primers, revealed the presence of the mutated and wild type *PTPN11* alleles in both BM and spleen of mouse#1, as well as in DNA from BM of mouse#2. DNA of the spleen of mouse#2 was not available. Mutant allele frequencies (MAF) of *PTPN11* were 50%, corresponding to heterozygous mutations in all cells (Supplementary Figure S3). Mutation analysis of *JAK3* and *SETBP1*, however, showed a different mutational pattern. Only 2 out of 3 mutations (the *SETBP1* mutation and only one of the 2 *JAK3* mutations) were identified in the BM and spleen of mouse #1, whereas the clone harboring the JAK3p.L857P mutation was undetectable, suggesting a distinctive propagation capacity of mutated JAK3 clones (Supplementary Figure S3). In the DNA of mouse#2 with positivity for the *PTPN11* mutation, *JAK3* and *SETBP1* mutations were not detected. The latter is suggestive of a reduced propagating capacity of cells lacking the secondary mutations, although the absence of BM engraftment in mouse#2 may also be ascribed to biological variability of the model (Supplementary Figure S3). In line with increased propagating capacity of clones with additional mutations, two recent papers by Stieglitz et al., [17] and Caye et al., [18] reported an association with worse outcome of JMML patients carrying > 1 mutation (secondary alterations). Our intriguing results of xenotransplanted JMML specimens are, however limited to a small number of mice, a problem frequently faced in studies of JMML patients where access to large biological samples is often a limiting factor.

Increased propagating capacity of clones harboring *SETBP1* mutations was suggested to involve transcriptional activation of the *HOXA9* [6]. In line with this notion, we found that JMML patients carrying a *SETBP1* mutation had increased expression of *HOXA9* as compared with patients harboring the wild-type protein (Supplementary Figure S4). Moreover, we found in mutated patients up-regulation of the RNA-binding protein encoded by *RPBMS* (Supplementary Figure S4), which physically interacts with SMAD proteins, key mediators of TGF-β signaling [19]. The over-expression of *RPBMS* enhances Smad-dependent transcriptional activity in a TGF-β-dependent manner, causing an alteration in the TGF-β pathway in patients with *SETBP1* mutations, as reported for aCML patients [9]; further studies are needed to understand the physiological activity of SETBP1 in JMML patients.

In conclusion, this study led to the identification of secondary mutations in *SETBP1* (10%) and *JAK3* (2, 8%) in a cohort of patients with JMML already present at diagnosis. The frequency of mutated *SETBP1* was slightly higher in the AIEOP cohort (10%) compared to the cohorts from Japan (6%) [12] and a COG study (7%) [5]. In the latter study, using ddPCR the frequency of *SETBP1* mutations increases 4-fold (30%) comprising a high number of patients with mutated sub-clones. In the AIEOP cohort, we identified two new mutations in the SETBP1 SKI domain.

The frequency of mutated *JAK3* was higher among JMML patients from Japan (10%) [12] compared with that (2.6%) detected in the AIEOP cohort. Similarly to the *SETBP1* mutation, also the frequency of *JAK3* mutations at diagnosis of JMML might increase using ddPCR, this technique being capable to detect also minor mutated sub-clones. The only transcribed *JAK3* mutation found in our study was tertiary to *PTPN11* and *SETBP1*. A prerequisite for the former mutations of *JAK3* to occur is, however, excluded, since in the study from Japan *JAK3* mutations occur also in combination with mutated *RAS* [12]. Our findings provide evidence for the secondary nature of *SETBP1* and *JAK3* mutations, although *SETBP1* mutations occurred in early cancer-initiating precursor cells, and increased propagating capacity of mutated clones maybe due to their role as transcription regulator. The effective role of these mutations in particular of *JAK3* in the pathogenesis of JMML remains to be elucidated.

## MATERIALS AND METHODS

### Patients

Seventy patients enrolled in the EWOG-MDS 2006 study, with a diagnosis of JMML were included in this study (Supplementary Table 1). Written informed consent was obtained from patients' parents or legal guardians before sample collection in accordance with the Declaration of Helsinki.

Diagnosis of JMML was based on the standard criteria proposed by the JMML Working Group during the second International JMML Symposium [20]. Presence of mutations of *PTPN11, RAS* and *CBL* was determined as part of the diagnostic work-up of all patients included in the study.

### Sequencing

Genomic DNA was extracted from total BM cells of patients at diagnosis of JMML using Gentra Puregene DNA Purification Kit (Qiagen, Monza, Italy) following the manufacturer's protocol.

All samples were amplified and sequenced with primers listed in Supplementary Table 2, covering the entire regions encoding for the SKI homologous domain of *SETBP1* and for the PTK domain of the *JAK3* gene, where the majority of mutations for both genes have been found. PCR amplification was performed using 50 ng of genomic DNA as template followed by bidirectional Sanger sequencing.

Total RNA was extracted using Trizol reagents (Invitrogen-Life Technologies Karlsruhe, Germany) and was reverse transcribed with SuperScript II system (Invitrogen-Life Technologies) using random hexamer primers, according to the manufacturer's instructions. Target sequences for *SETBP1* and *JAK3* were PCR amplified and sequenced by Sanger sequencing.

### Xenograft mouse model

Mononuclear cells (1 × 10^7^ per transplant) from the spleen of a JMML patient carrying a *PTPN11* driver mutation who underwent splenectomy were intravenously injected into NSG (NOD.Cg-Prkdc^scid^ Il2rg^tm1Wjl^/Sz) mice. Animal experiments were approved by the appropriate authorities. After transplantation, mice were regularly examined for human cell load by flow cytometry gating for huCD45 and huCD33. After 9 months, mice were sacrificed and cells were harvested from BM and spleen. DNA was analyzed for *PTPN11* mutations by ultra-deep sequencing with 454 GS Junior System using human *PTPN11* specific fusion primers with adaptor and MID as requested by the manufacturer (Roche Applied Science, Penzberg, Germany).

### Colony assay

Epstein–Barr virus-induced B-lymphoblastoid cell line (B-LCL), phytohemagglutinin (PHA)-activated T-lymphoblastoid cells (PHA-activated T cells), erythroid burst-forming units (BFU-E), CFU-GM, and LTC-IC colonies were obtained as previously reported [16].

### Statistical analysis

To compare the frequency of mutations with that of other clinical and biological parameters, categorical variables were analyzed using Fisher's exact test, while continuous variables were analyzed with Mann-Whitney *U* test. Probabilities of overall survival (OS) were calculated by the Kaplan-Meier method and compared with the Log-Rank test using Bioconductor packages (www.r-project.org).

### Gene expression analysis

Total RNA was extracted using Trizol, and quality was checked by the use of Agilent 2100 Bioanalyzer. Microarray data were obtained using 3′ IVT Express Kit and HG-U133 Plus 2.0 array (Affymetrix, Santa Clara, CA, USA). Gene expression data of this study were part of a previous study deposited in GSE14858 [21]. For this study we selected samples that had been screened for *SETBP1* mutations; p-values were determined using Welch's *t*-test.

## SUPPLEMENTARY MATERIALS FIGURES AND TABLES


