# Supplementary material for: Mutations of SETBP1 and JAK3 in juvenile myelomonocytic leukemia: a report from the Italian AIEOP study group
Source: Oncotarget. 2016 Mar 9;7(20):28914–9. doi: 10.18632/oncotarget.8016 (PMC5045366; doi:10.18632/oncotarget.8016)
Supplement: Supplementary file 1 [file oncotarget-07-28914-s001.pdf]

Mutations of *SETBP1* and *JAK3* in juvenile myelomonocytic leukemia: a report from the italian AIEOP study group

Supplementary Materials

Supplementary Table S1: List of mutations in JMML patients

Patient	Driver mutations	*SETBP1* and *JAK3* mutations	SIFT	PolyPhen	MutationTaster
**Ptl**	*KRAS*	wt	-	-	-
**Pt2**	*No Mutation*	wt	-	-	-
**Pt3**	*PTPN11*	wt	-	-	-
**Pt4**	*No Mutation*	wt	-	-	-
**Pt5**	*NF1*	wt	-	-	-
**Pt6**	*PTPN11*	wt	-	-	-
**Pt7**	*PTPN11*	wt	-	-	-
**Pt8**	*No Mutation*	wt	-	-	-
**Pt9**	*PTPN11*	wt	-	-	-
**Pt10**	*NRAS*	wt	-	-	-
**Pt11**	*PTPN11*	wt	-	-	-
**Ptl2**	*NRAS*	wt	-	-	-
**Pt13**	*No Mutation*	wt	-	-	-
**Pt14**	*KRAS*	wt	-	-	-
**Pt15**	*PTPN11*	wt	-	-	-
**Pt16**	*PTPN11*	wt	-	-	-
**Pt17**	*PTPN11*	wt	-	-	-
**Pt18**	*PTPN11*	wt	-	-	-
**Pt19**	*KRAS*	wt	-	-	-
**Pt20**	*NRAS*	wt	-	-	-
**Pt21**	*NRAS*	wt	-	-	-
**Pt22**	*ND*	wt	-	-	-
**Pt23**	*PTPN11*	wt	-	-	-
**Pt24**	*PTPN11*	wt	-	-	-
**Pt25**	*PTPN11*	wt	-	-	-
**Pt26**	*NRAS*	wt	-	-	-
**Pt27**	*PTPN11*	wt	-	-	-

**Pt28**	*PTPN11*	wt	-	-	-
**Pt29**	*No Mutation*	wt	-	-	-
**Pt30**	*KRAS*	wt	-	-	-
**Pt31**	*CBL*	wt	-	-	-
**Pt32**	*CBL*	wt	-	-	-
**Pt33**	*No Mutation*	wt	-	-	-
**Pt34**	*PTPN11*	wt	-	-	-
**Pt35**	*KRAS*	wt	-	-	-
**Pt36**	*CBL Heterozygote*	wt	-	-	-
**Pt37**	*KRAS*	wt	-	-	-
**Pt38**	*No Mutation*	wt	-	-	-
**Pt39**	*PTPN11*	wt	-	-	-
**Pt40**	*KRAS*	wt	-	-	-
**Pt41**	*NRAS*	wt	-	-	-
**Pt42**	*NRAS*	wt	-	-	-
**Pt43**	*PTPN11*	wt	-	-	-
**Pt44**	*KRAS*	wt	-	-	-
**Pt45**	*KRAS*	wt	-	-	-
**Pt46**	*NRAS*	wt	-	-	-
**Pt47**	*KRAS*	wt	-	-	-
**Pt48**	*PTPN11*	wt	-	-	-
**Pt49**	*PTPN11*	wt	-	-	-
**Pt50**	*ND*	wt	-	-	-
**Pt51**	*PTPN11*	wt	-	-	-
**Pt52**	*NRAS*	wt	-	-	-
**Pt53**	*NRAS*	wt	-	-	-
**Pt54**	*PTPN11*	wt	-	-	-
**Pt55**	*PTPN11*	wt	-	-	-
**Pt56**	*PTPN11*	wt	-	-	-
**Pt57**	*ND*	wt	-	-	-
**Pt58**	*PTPN11*	wt	-	-	-
**Pt59**	*NRAS*	wt	-	-	-
**Pt60**	*KRAS*	wt	-	-	-
**Pt61**	*PTPN11*	wt	-	-	-
**Pt62**	*No Mutation*	wt	-	-	-
**Pt63**	*NRAS*	*SETBP1* G870S	Deleterious	Probably Damaging	Dgisease causing
**Pt64**	*PTPN11*	*SETBP1* G870S	Deleterious	Probably Damaging	Dgisease causing
**Pt65**	*PTPN11*	*SETBP1* S867I	Deleterious	Probably Damaging	Dgisease causing
**Pt66**	*PTPN11*	*JAK3* E958K	Deleterious	Probably Damaging	Dgisease causing

**Pt67**	*KRAS*	*SETBP1* D868N	Deleterious	Probably Damaging	Polymorphism
**Pt68**	*PTPN11*	*SETBP1* G870S	Deleterious	Probably Damaging	Disease causing
**Pt69**	*PTPN11*	*SETBP1* D868N, *JAK3* L857P,L857Q	Deleterious	Probably Damaging	Disease causing
**Pt70**	*NRAS*	*SETBP1* P855S	Deleterious	Probably Damaging	Disease causing

Supplementary Table S2: Primers sequences

Gene	Forward_Primer	Reverse_Primer
**SETBP1_SKIdomain**	CAAAT CT CCAGCCCAT CAGT	TTCTCCGCTTGGTCAGAAGT
**JAK3_exon15-16_Genomic**	GCCAAACAGACTCTTCATTCATC	GGAT CAGGGAT CCACTT CCT
**JAK3_exon17_Genomic**	AGATTGGGGT GGGT CTATT G	ACCACGCT CCTTCCACT G
**JAK3_exon18-19_Genomic**	GAGGTT GCACAGCAAGT CAA	GCAGGAGGGTAAGAATGTGC
**JAK3_exon21_Genomic**	GT CACGCTTGGGGTACCT G	CTGGGGAGCAAAGCAGC
**JAK3_exon15-17_cDNA**	T GGT CAAACAGCT GGCCTAC	AGGTGTGGGGTCTGAGAGGA
**JAK3_exon18-21_cDNA**	AACTTT GGCAGCGT GGAG	TACCAGAAAATGGGGCTCTG

Supplementary Table S3: Screening of hematopoietic cell colonies in two patients harboring *PTPN11* and *SETBP1* mutations

	**#Pt65**		**#Pt64**	
	PTPN11	***SETBP1***	***PTPN11***	***SETBP1***
	c.215C > T	c.2600G > T	c.215C > T	c.2608G > A
**PBMC**	Positive	Positive	Positive	Positive
**LTC-IC**	Positive	ND	Positive	Positive
**BFU-E**	ND	Positive	Positive	Positive
**CFU-GM**	Positive	Positive	Positive	Positive
**PHA-activated T cells**	Positive	Negative	Negative	ND
**B-LCL**	Positive	Positive	Negative	ND

Positive means detection of mutations by Sanger sequencing and negative absence of mutations; ND sequencing not done; PBMC: peripheral blood mononuclear cells; LTC-IC: long-term culture initiating cell; BFU-E: Burst forming erythroid unit; CFU-GM: colony forming unit granulocyte macrophage; *PHA: phytohemagglutin*; B-LCL: B-lymphoblastoid cell lines.

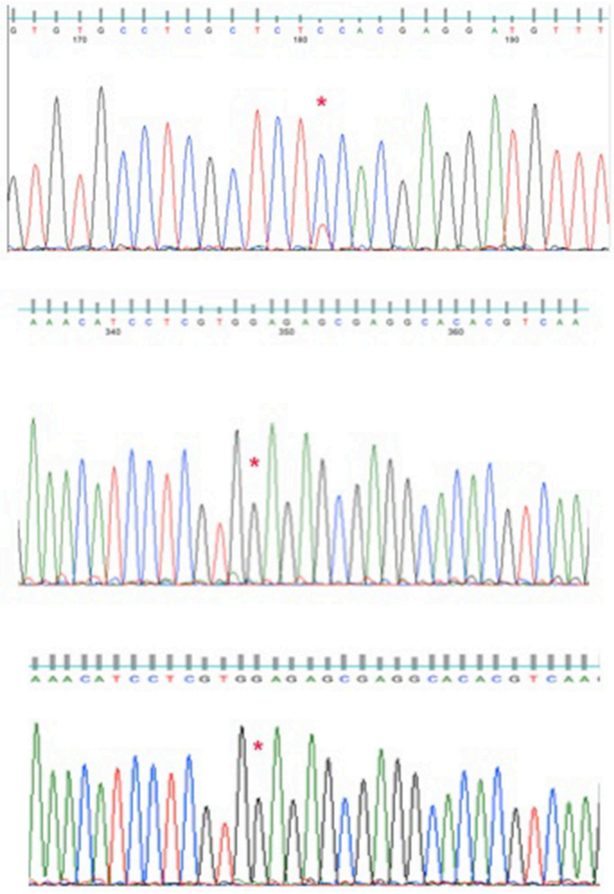

Supplementary Figure S1: Results of Sanger sequencing reported for patient #Pt66 on *JAK3* gene. The upper panel showed the presence of the mutation found in the DNA of the patient at diagnosis. Second and third panel showed for the same patient the absence of the mutation after sequencing of cDNA at diagnosis and at relapse, respectively.

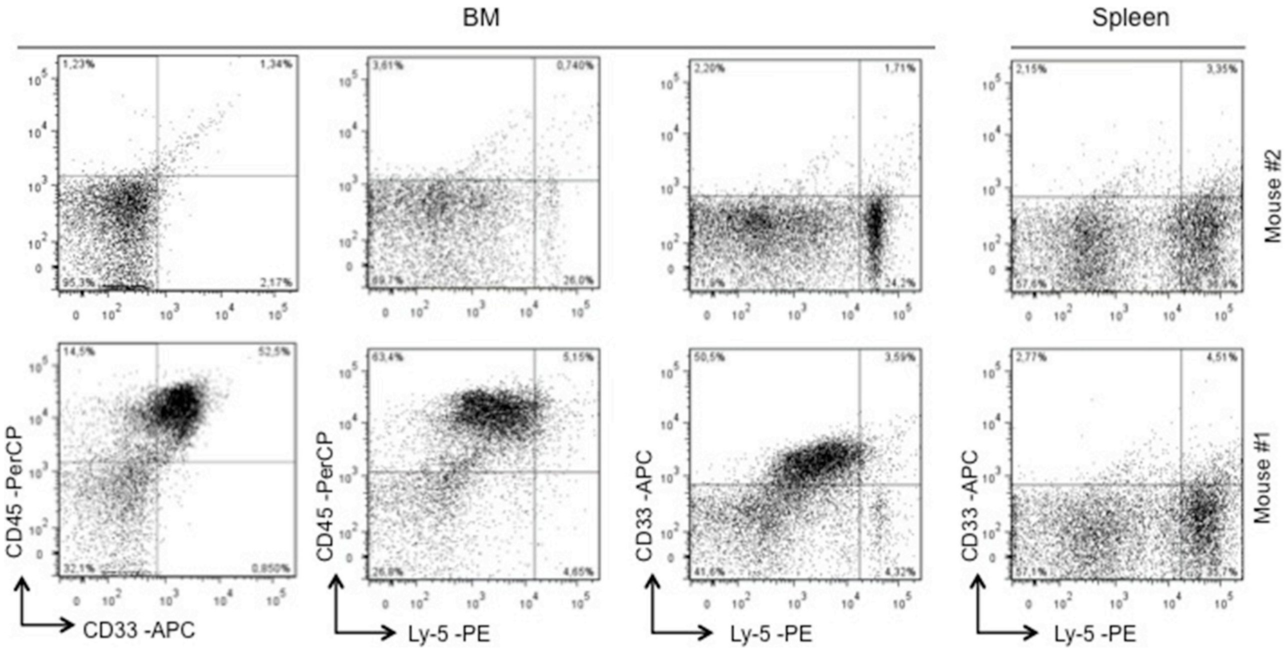

Supplementary Figure S2: Flow cytometry analysis of engrafted mice. Cells from bone marrow and spleen from two mice were analyzed with cell surface markers specific for human cells (huCD45, huCD33) and mouse (Ly-5). Upper panel showed percentage of engrafted human cells in bone marrow and spleen for mouse#2, bottom panel for mouse #1.

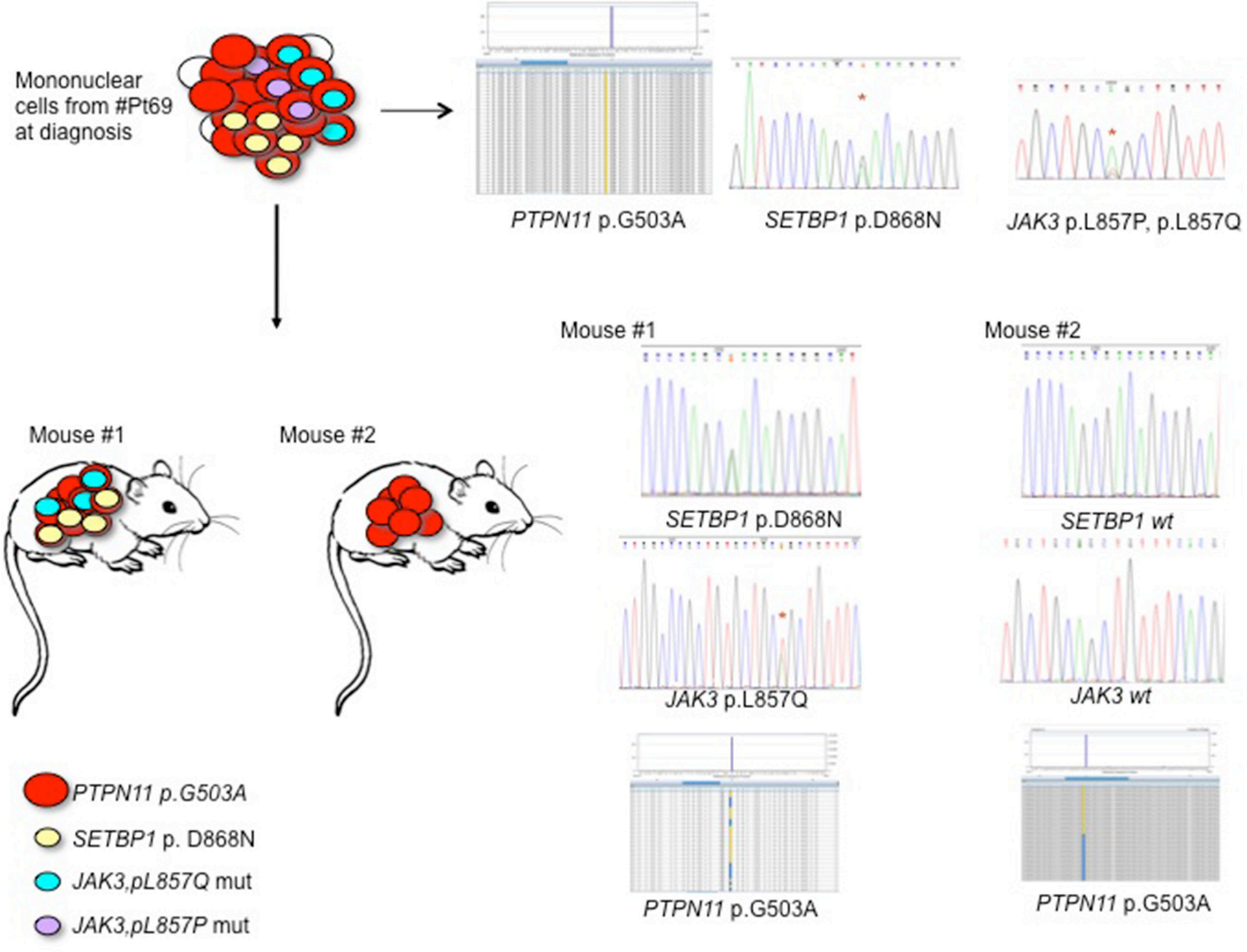

Supplementary Figure S3: Clonal selection of *SETBP1* and *JAK3* mutations after xenotransplantation. Mononuclear cells of patient #Pt69 harboring mutated *PTPN11* mutation and additional secondary genetic alterations in *SETBP1* and *JAK3* (two different subclones), were xenotransplanted in NSG mice. Sequence analysis of engrafted cells revealed clonal selection of the additional mutations. As shown in the panel mouse#1 presented *PTPN11*, *SETBP1* and only the *JAK3* L857Q mutation; mouse #2 harbored just the *PTPN11* mutation.

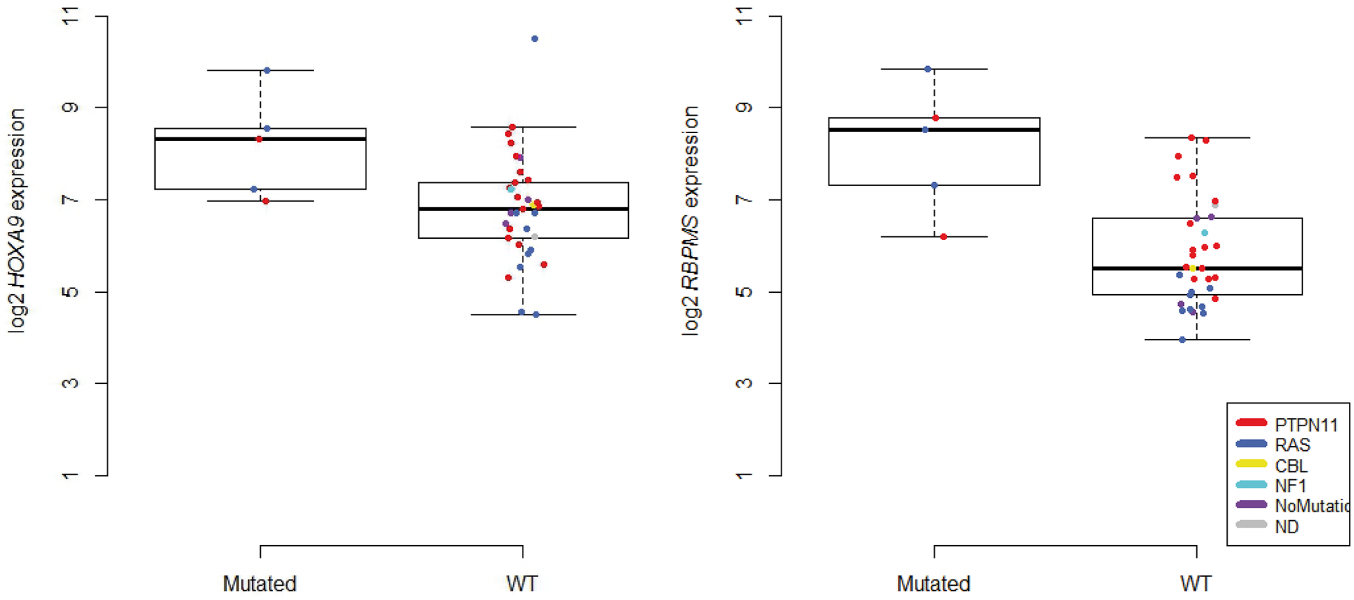

Supplementary Figure S4: Box plots showing the differences in *HOXA9* and *RPBMS* gene expression among JMML patients. Patients were divided according to the mutational status of *SETBP1*. Expression values in log2 (y-axes) derived from Affymetrix platform array. *P*-value was obtained using Welch *t*-test ($p = 0.05$ for *HOXA9* and $p = 0.018$ for *RBPMS*).

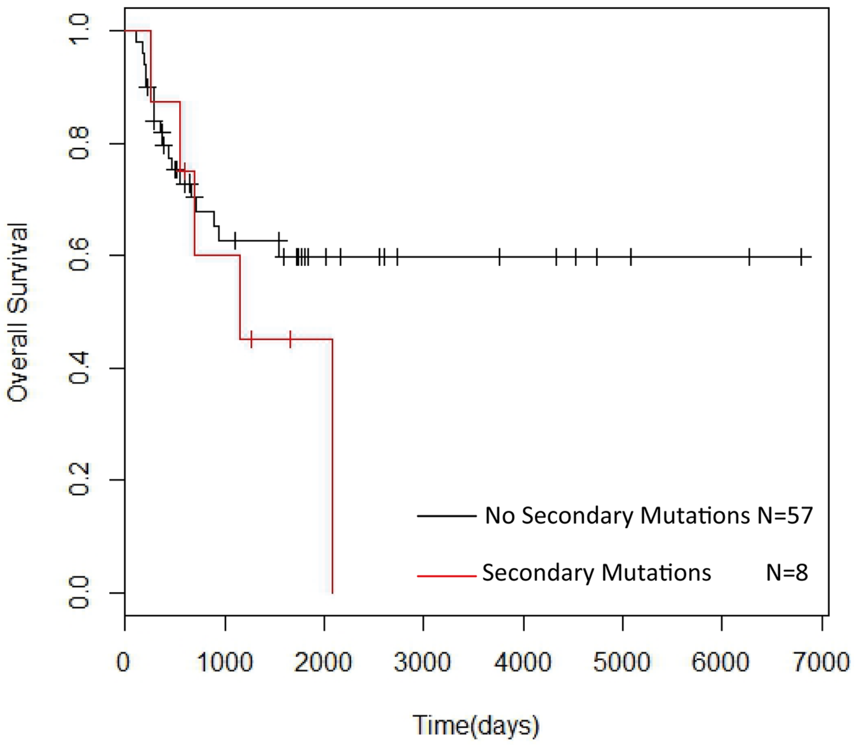

Supplementary Figure S5: Probability of overall survival from diagnosis of the JMML patients stratified by presence of secondary mutations in *SETBP1* and *JAK3* or absence of these mutations. (log-rank test $p = 0.3$).
